# Net clinical benefit of antithrombotic therapy for atrial fibrillation patients with stable coronary artery disease

**DOI:** 10.3389/fcvm.2022.991293

**Published:** 2022-08-22

**Authors:** So-Ryoung Lee, Jin-Hyung Jung, Eue-Keun Choi, Seung-Woo Lee, Soonil Kwon, Ji-Suck Park, Jeehoon Kang, Kyung-Do Han, Kyung Woo Park, Seil Oh, Gregory Y. H. Lip

**Affiliations:** ^1^Department of Internal Medicine, Seoul National University Hospital, Seoul, South Korea; ^2^Department of Medical Statistics, College of Medicine, Catholic University of Korea, Seoul, South Korea; ^3^Department of Internal Medicine, Seoul National University College of Medicine, Seoul, South Korea; ^4^Department of Statistics and Actuarial Science, Soongsil University, Seoul, South Korea; ^5^Liverpool Centre for Cardiovascular Science, University of Liverpool and Liverpool Chest and Heart Hospital, Liverpool, United Kingdom; ^6^Department of Clinical Medicine, Aalborg University, Aalborg, Denmark

**Keywords:** atrial fibrillation, coronary artery disease, antithrombotic therapy, oral anticoagulant, antiplatelet agent

## Abstract

**Objectives:**

To compare the net clinical benefit of oral anticoagulant (OAC) monotherapy to OAC plus single antiplatelet therapy (SAPT) in patients with atrial fibrillation (AF) and stable coronary artery disease (CAD) at 1- and 3-year after percutaneous coronary intervention (PCI).

**Background:**

It has not been studied whether the net clinical benefit of the antithrombotic treatment options differs depending on the elapsed time from the index PCI.

**Methods:**

Using the Korean nationwide claims database, we included AF patients who underwent PCI from 2009 to 2019 and constructed two cohorts: 1- and 3-year after PCI. In each cohort, the baseline characteristics of two groups were balanced using propensity score weighting. Ischemic stroke, myocardial infarction, major bleeding, and composite clinical outcomes were analyzed.

**Results:**

Among patients with 1-year after PCI, OAC monotherapy (*n* = 678), and OAC plus SAPT (*n* = 3,159) showed comparable results for all clinical outcomes. In patients with 3-year after PCI, OAC monotherapy (*n* = 1,038) and OAC plus SAPT (*n* = 2,128) showed comparable results for ischemic stroke and myocardial infarction, but OAC monotherapy was associated with a lower risk of composite clinical outcomes (HR 0.762, 95% CI 0.607–0.950), mainly driven by the reduction of major bleeding risk (HR 0.498, 95% CI 0.345–0.701).

**Conclusion:**

Oral anticoagulant monotherapy may be a comparable choice for patients with AF and stable CAD compared to OAC plus SAPT. In patients with stable CAD more than 3-year after index PCI, OAC monotherapy would be a better choice, being associated with less major bleeding and a positive net clinical benefit.

## Introduction

Oral anticoagulant (OAC) monotherapy is generally recommended in patients with atrial fibrillation (AF) and stable coronary artery disease (CAD) (1–4). In a previous meta-analysis, OAC monotherapy showed a comparable risk of major adverse cardiovascular events and a lower risk of major bleeding than OAC plus single antiplatelet agent (SAPT) (5). There have also been two randomized clinical trials that evaluated the optimal antithrombotic therapy for patients with AF and stable CAD (6, 7). The OAC-ALONE trial was the first randomized trial comparing OAC monotherapy vs. OAC plus SAPT in patients with AF and stable CAD beyond 1-year after undergoing percutaneous coronary intervention (PCI) (6). However, non-inferiority of OAC monotherapy to OAC plus SAPT for the composite of major adverse cardiovascular events was not established because of inadequate statistical power (6). Recently, the AFIRE trial showed that rivaroxaban monotherapy was non-inferior for efficacy and superior for safety to rivaroxaban plus SAPT in patients with AF and stable CAD (7).

Although the AFIRE trial demonstrated that rivaroxaban monotherapy is superior to rivaroxaban plus SAPT in primary safety outcomes, there have been conflicting data regarding the comparative effectiveness and safety of OAC monotherapy vs. OAC plus SAPT according to the time from index PCI to study enrollment (8, 9). Considering the temporal dynamic of the risk of stent thrombosis after PCI and thromboembolic risk in patients with AF (10), we can hypothesize that the efficacy and safety of antithrombotic treatment strategies can temporally vary. However, it has not been studied whether the net clinical benefit of the antithrombotic treatment options differs depending on the elapsed time from the index PCI.

In this study, we aimed to compare the effectiveness, safety, and net clinical benefit of OAC monotherapy to OAC plus SAPT in patients with AF and stable CAD at 1- and 3-year after PCI in a contemporary real-world observational cohort.

## Materials and methods

### Data source, study design, and study population

This analysis was performed based on the Korean nationwide claims database from the Korean Health Insurance Review Agency (HIRA) database. In South Korea, all citizens are subscribed to the medical insurance system, called the Korean National Health Insurance Service (NHIS) provided by the Korean government (11). Information on subscribers’ medical use is collected for NHIS operation, and information on medical use, which becomes insurance coverage, is submitted from health care providers. The submitted information is reviewed by the Korean HIRA, which is a quality control department that provides a review of the medical costs incurred. The Korean HIRA database contains all medical expenses claim data of the entire Korean population, including subscribers’ demographic information, diagnoses, examinations, prescriptions, and procedures for both inpatient and outpatient services (11, 12). Diagnoses were coded based on the International Classification of Disease, Tenth Revision, Clinical Modification (ICD-10-CM) codes (11, 12).

**FIGURE 1:**
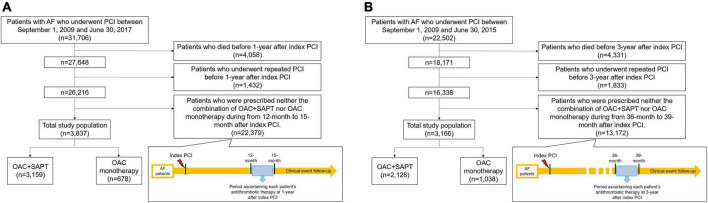
Study design and patient enrollment flow. **(A)** Cohort 1: patients with AF who underwent PCI, 1-year after index PCI. **(B)** Cohort 2: patients with AF who underwent PCI, 3-year after index PCI. AF, atrial fibrillation; OAC, oral anticoagulant; PCI, percutaneous coronary intervention; SAPT, single antiplatelet agent.

Using the Korean nationwide claims database, we included AF patients who underwent PCI from January 1, 2009 to February 28, 2019. Considering dynamic changes in antithrombotic therapy according to the period after receiving PCI, the index antithrombotic treatment was independently defined at different times after receiving PCI and we constructed two cohorts: 1- and 3-year after PCI (Figure 1). Cohort 1 consisted of patients who had just passed 1 year after PCI. Patients with AF who underwent PCI between September 1, 2009 and June 30, 2017, were firstly identified. Patients who died before 1-year after PCI and underwent repeated PCI before 1-year after PCI were excluded. Among these, the OAC monotherapy group and OAC plus SAPT group were defined by identifying prescriptions between 12 and 15 months from PCI (Figure 1A). Cohort 2 was defined as patients 3 years after PCI. Patients with AF who underwent PCI between September 1, 2009 and June 30, 2015, were included. Similar to cohort 1, patients who died before 3-year after PCI and underwent repeated PCI before 3-year after PCI were excluded. OAC monotherapy group and OAC plus SAPT group were identified by the prescription between 36 and 39 months from PCI (Figure 1B).

The study design was approved by the Institutional Review Board of the Seoul National University Hospital (E-1911-052-1078). The review board waived informed consent since each patient is de-identified and encrypted in the HIRA database to ensure patient privacy.

### Covariates

Subjects’ age, sex, comorbidities including hypertension, diabetes, dyslipidemia, heart failure, prior myocardial infarction, peripheral artery disease, prior ischemic stroke/transient ischemic attack/systemic embolism, prior intracranial hemorrhage, prior gastrointestinal bleeding, renal disease, and liver disease were ascertained by the prespecified operational definitions summarized in Supplementary Table 1 (13, 14). Concomitant medications include renin-angiotensin-aldosterone system inhibitors, beta-blockers, calcium channel blockers, loop diuretics, statins, non-steroidal anti-inflammatory drugs, and proton-pump inhibitors were ascertained based on the prescription records. The type of OAC [warfarin or direct oral anticoagulant (DOAC) including rivaroxaban, dabigatran, apixaban, and edoxaban], dose of DOAC, type of antiplatelet agents among aspirin, clopidogrel, prasugrel or ticagrelor were also identified. CHA_2_DS_2_-VASc score and modified HAS-BLED score were calculated by the operational definitions of comorbidities and medical history including concomitant medication (Supplementary Table 1) (13, 14).

### Study outcomes and follow-up

During the follow-up period, composites of ischemic stroke and myocardial infarction occurrence were identified for effectiveness evaluation. For safety evaluation, major bleeding was defined as a composite of intracranial hemorrhage, gastrointestinal bleeding, and extracranial/unclassified major bleeding. We identified the major bleeding that occurred during the follow-up period. To assess net clinical benefit, composite clinical outcomes of ischemic stroke, myocardial infarction, and major bleeding were ascertained. Furthermore, we reported each component of effectiveness and safety outcome as follows: ischemic stroke, myocardial infarction, intracranial hemorrhage, gastrointestinal bleeding, and gastrointestinal bleeding requiring transfusion. Clinical outcomes were defined by the ICD-10-CM codes and detailed definitions of clinical outcomes are summarized in Supplementary Table 1.

To evaluate the accuracy of the operational definitions of clinical outcomes including ischemic stroke, myocardial infarction, intracranial hemorrhage, and gastrointestinal bleeding, we conducted a validation study in a tertiary hospital with 200 randomly chosen patients with the relevant ICD-10-CM codes for each event (15). Patients’ medical records were reviewed by two physicians (JP and SK). The positive predictive values of the operational definitions were 91.2, 92, 95.1, and 91.7% for ischemic stroke, myocardial infarction, intracranial hemorrhage, and gastrointestinal bleeding (15). In each cohort, the index date was the first date of OAC monotherapy or OAC plus SAPT prescription. Patients were censored at the outcome events or the end of the study period (February 28, 2019), whichever came first.

### Statistical analysis

Continuous variables are presented as mean (standard deviation) and median (interquartile ranges, IQR). Categorical variables are presented as number and percentage. For each clinical outcome, the crude incidence rate for each clinical outcome was estimated by dividing the number of incidents during the follow-up period by the number of 100 person-years at risk. Unadjusted hazard ratios (HRs) and 95% confidence intervals (CIs) were analyzed for estimation of the risk of clinical outcomes using the Cox proportional hazards regression models.

To compare the OAC monotherapy and OAC plus SAPT groups, propensity score methods were used (16). A logistic regression model with all baseline factors (except for DOAC dose) was used to determine the probability score for being in each treatment group. To balance baseline characteristics across the two treatment groups, time-to-event analyses were conducted using inverse probability of treatment weighting (IPTW) analysis with stabilized weights computed from the propensity score (17, 18). Following IPTW, the covariate balance between the two groups was determined using the absolute standardized difference (ASD) (19). In a covariate, an ASD of ≤0.1 (10%) indicated that the two groups were well-balanced, with a negligible difference. The weighted number of events throughout the follow-up period was divided by 100 person-years at risk to calculate the weighted incidence rates. Survival analysis with the Kaplan-Meier method (log-rank test) and weighted Cox proportional hazards regression models with IPTW were used to determine the risk of clinical outcomes for OAC monotherapy and OAC plus SAPT (reference).

To provide complementary results, we conducted multivariable Cox analyses for a sensitivity analysis. Age, sex, hypertension, diabetes mellitus, dyslipidemia, heart failure, prior myocardial infarction, peripheral artery disease, prior stroke/transient ischemic attack/systemic embolism, prior intracranial hemorrhage, prior gastrointestinal bleeding, renal disease, liver disease, CHA_2_DS_2_-VASc score, modified HAS-BLED score, and OAC type (warfarin or DOAC) were included for the multivariable-adjusted Cox analyses.

SAS software, version 9.3 (SAS Institute, Cary, NC, United States), was used for all statistical analyses, and a two-tailed *p*-value of 0.05 was considered statistically significant.

## Results

In cohort 1 among patients 1-year after PCI, 678 patients with OAC monotherapy and 3,159 patients with OAC plus SAPT were included. In cohort 2 among patients 3-years after PCI, 1,038 patients with OAC monotherapy and 2,128 patients with OAC plus SAPT were enrolled.

Baseline characteristics are presented in Tables 1, 2. In cohort 1, the OAC monotherapy group were older, more likely to be women, and had higher CHA_2_DS_2_-VASc scores than OAC plus SAPT group. OAC monotherapy group showed a higher prevalence of prior ischemic stroke/transient ischemic attack/systemic embolism than the OAC plus SAPT group. Regarding OAC types, the OAC monotherapy group was more likely to be prescribed DOAC rather than warfarin compared to OAC plus SAPT group. Among DOAC users, the OAC plus SAPT group was more likely to be prescribed a reduced dose of DOAC than the OAC monotherapy group. Among SAPT for OAC plus SAPT group, clopidogrel was the most commonly prescribed (65.9%), followed by aspirin (33.8%). In cohort 2, similar differences between the two groups were observed as in cohort 1. OAC monotherapy group were older, more likely to be women, and had higher CHA_2_DS_2_-VASc scores compared to OAC plus SAPT group. Diabetes mellitus was more prevalent in OAC plus SAPT group than in the OAC monotherapy group. DOAC prescription was more common in the OAC monotherapy group. Among DOAC users, reduced dose DOAC use was more common in patients with OAC plus SAPT. Among SAPT for OAC plus SAPT group, aspirin was the most commonly prescribed (52.2%), followed by clopidogrel (47.7%). The baseline characteristics were well-balanced after IPTW between the two groups in both cohorts except for the DOAC dose (Tables 1, 2 and Supplementary Figure 1).

**TABLE 1:** Baseline characteristics of oral anticoagulant (OAC) plus single antiplatelet therapy (SAPT) and OAC monotherapy groups at 1-year after index percutaneous coronary intervention (PCI).

	Before IPTW			Post IPTW		
	OAC + SAPT (*n* = 3,159)	OAC monotherapy (*n* = 678)	ASD	OAC + SAPT (*n* = 3,159)	OAC monotherapy (*n* = 676)	ASD
The year of index PCI						
2009	122 (3.9)	33 (4.9)		119 (3.8)	35 (5.2)	
2010	194 (6.1)	44 (6.5)		189 (6.0)	50 (7.4)	
2011	178 (5.6)	40 (5.9)		172 (5.5)	47 (6.9)	
2012	237 (7.5)	50 (7.4)		232 (7.3)	55 (8.1)	
2013	291(9.2)	63 (9.3)		283 (8.9)	72 (10.6)	
2014	429(13.6)	70 (10.3)		426 (13.5)	69 (10.3)	
2015	503(15.9)	104 (15.3)		508 (16.1)	98 (14.5)	
2016	742(23.5)	172 (25.4)		758 (24.0)	158 (23.4)	
2017	463(14.7)	102 (15.0)		474 (15.0)	92 (13.6)	
Age, years			0.068			0.005
Mean (SD)	70.4 ± 9	71.03 ± 9.32		70.51 ± 9.05	70.46 ± 9.14	
Median (IQR)	72 (65–77)	72 (65–78)		72 (65–77)	71 (65–77)	
Age group						
<65 years	733 (23.2)	159 (23.5)		728 (23.1)	162 (24.0)	
65–74 years	1323 (41.9)	242 (35.7)		1295 (41.0)	269 (39.8)	
≥75 years	1103 (34.9)	277 (40.9)		1136 (36.0)	245 (36.3)	
Women	1029 (32.6)	264 (38.9)	0.133	1065 (33.7)	229 (33.9)	0.003
Comorbidities						
Hypertension	2873 (91.0)	619(91.3)	0.012	2875 (91.0)	614 (90.8)	0.006
Diabetes mellitus	1225 (38.8)	249 (36.7)	0.042	1213 (38.4)	255 (37.7)	0.013
Dyslipidemia	2621 (83.0)	547 (80.7)	0.059	2608 (82.6)	558 (82.5)	0.001
Heart failure	1451 (45.9)	339 (50.0)	0.081	1475 (46.7)	320 (47.3)	0.011
Prior myocardial infarction	1075 (34.0)	221 (32.6)	0.030	1068 (33.8)	231 (34.1)	0.007
Peripheral artery disease	797 (25.2)	170 (25.1)	0.003	798 (25.2)	173 (25.6)	0.008
Prior ischemic stroke/TIA/SE	632 (20.0)	167 (24.6)	0.111	658 (20.8)	141 (20.8)	0.000
Prior intracranial hemorrhage	16 (0.5)	9 (1.3)	0.086	21 (0.7)	4.7 (0.7)	0.004
Prior gastrointestinal bleeding	204 (6.5)	57 (8.4)	0.074	215 (6.8)	47 (6.9)	0.005
Renal disease	458 (14.5)	100 (14.8)	0.007	460 (14.5)	98 (14.5)	0.001
Liver disease	1049 (33.2)	218 (32.2)	0.022	1042 (33.0)	219 (32.3)	0.014
CHA_2_DS_2_-VASc score			0.159			0.004
Mean (SD)	3.69 ± 1.78	3.97 ± 1.81		3.74 ± 1.8	3.75 ± 1.78	
Median (IQR)	3 (2–5)	4 (3–5)		4 (2–5)	4 (2–5)	
Modified HAS-BLED			0.050			0.005
Mean (SD)	3.33 ± 0.94	3.38 ± 0.97		3.34 ± 0.95	3.33 ± 0.95	
Median (IQR)	3 (3–4)	3 (3–4)		3 (3–4)	3 (3–4)	
Concomitant medications						
RAAS inhibitors	2612 (82.7)	552 (81.4)	0.033	2111 (66.8)	449 (66.3)	0.010
Beta-blockers	2682 (84.9)	572 (84.4)	0.014	2685 (85.0)	571 (84.4)	0.014
Calcium channel blockers	2211 (70.0)	476 (70.2)	0.004	2213 (70.1)	468 (69.3)	0.017
Loop diuretics	1875 (59.4)	432 (63.7)	0.089	1887 (59.7)	423 (62.5)	0.057
Statins	2787 (88.2)	579 (85.4)	0.083	2783 (88.1)	585 (86.5)	0.046
NSAID	2106 (66.7)	458 (67.6)	0.018	2111 (66.8)	449 (66.3)	0.010
Proton pump inhibitors	1595 (50.5)	339 (50)	0.009	1612 (51.0)	32 (47.5)	0.070
Antithrombotic therapy						
OAC type			0.191			0.004
Warfarin	1718(54.38)	304(44.84)		1665(52.7)	355 (52.5)	
DOAC	1441(45.62)	374(55.16)		1494 (47.3)	322 (47.5)	
DOAC dose			0.320			0.355
Standard dose DOAC	320 (22.2)	137 (36.6)		329 (22.0)	122.3(38.04)	
Reduced dose DOAC	1121 (77.8)	237 (63.4)		1166 (78.0)	199 (62.0)	
DOAC type						
Rivaroxaban	565 (17.9)	149 (22.0)		585 (18.5)	131 (19.3)	
Dabigatran	260 (8.2)	69 (10.2)		271 (8.6)	58 (8.6)	
Apixaban	432 (13.7)	106 (15.6)		448 (14.2)	89 (13.1)	
Edoxaban	184 (5.8)	50 (7.4)		190 (6.0)	44 (6.5)	
Antiplatelet agent type						
Aspirin	1069 (33.8)	0 (0)		1063 (33.7)	0 (0)	
Clopidogrel	2081 (65.9)	0 (0)		2087 (66.1)	0 (0)	
Prasugrel or ticagrelor	9 (0.3)	0 (0)		9.2 (0.3)	0 (0)	

IQR, interquartile ranges; DOAC, direct oral anticoagulant; IPTW, inverse probability of treatment weighting; NSAID, non-steroidal anti-inflammatory drug; OAC, oral anticoagulant; RAAS, renin-angiotensin-aldosterone system; SD, standard deviation; SE, systemic embolism; TIA, transient ischemic attack.

**TABLE 2:** Baseline characteristics of OAC plus SAPT and OAC monotherapy groups at 3-year after index PCI.

	Before IPTW			Post IPTW		
	OAC + SAPT (*n* = 2128)	OAC monotherapy (*n* = 1038)	ASD	OAC + SAPT (*n* = 2129)	OAC monotherapy (*n* = 1036)	ASD
The year of index PCI						
2009	225 (10.6)	78 (7.5)		210 (9.9)	89 (8.6)	
2010	242 (11.4)	95 (9.2)		227 (10.7)	106 (10.3)	
2011	266 (12.5)	112 (10.8)		248 (11.6)	132 (12.7)	
2012	304 (14.3)	130 (12.5)		297 (14.0)	134 (12.9)	
2013	369 (17.3)	206 (19.9)		386 (18.1)	203 (19.6)	
2014	471 (22.1)	251 (24.2)		494 (23.2)	222 (21.4)	
2015	251 (11.8)	166 (16.0)		267 (12.6)	151 (14.6)	
Age, years			0.185			0.001
Mean (SD)	68.06 ± 9.07	69.71 ± 8.74		68.61 ± 9.09	68.6 ± 8.85	
Median (IQR)	69 (63–74)	71 (65–76)		70 (64–75)	70 (63–75)	
Age group						
<65 years	645 (30.3)	256 (24.7)		603 (28.3)	299 (28.9)	
65–74 years	969 (45.5)	439 (42.3)		944 (44.3)	454 (43.8)	
≥75 years	514 (24.2)	343 (33.0)		582 (27.3)	283 (27.3)	
Women	595 (28.0)	385 (37.1)	0.195	660 (31.0)	321 (31.0)	0.000
Comorbidities						
Hypertension	1911 (89.8)	948 (91.3)	0.052	1924 (90.4)	940 (90.7)	0.011
Diabetes mellitus	799 (37.6)	328 (31.6)	0.125	757 (35.6)	370 (35.7)	0.002
Dyslipidemia	1698 (79.8)	834 (80.4)	0.013	1704 (80.1)	832 (80.3)	0.005
Heart failure	860 (40.4)	430 (41.4)	0.020	865 (40.6)	419 (40.4)	0.004
Prior myocardial infarction	691 (32.5)	327 (31.5)	0.020	684 (32.1)	331 (31.9)	0.004
Peripheral artery disease	511 (24.0)	247 (23.8)	0.005	513 (24.1)	250 (24.2)	0.001
Prior ischemic stroke/TIA/SE	417 (19.6)	206 (19.9)	0.006	422 (19.8)	206 (19.9)	0.002
Prior intracranial hemorrhage	13 (0.6)	8 (0.8)	0.019	14 (0.6)	6 (0.6)	0.002
Prior gastrointestinal bleeding	134 (6.3)	77 (7.4)	0.044	144 (6.7)	70 (6.7)	0.000
Renal disease	244 (11.5)	123 (11.9)	0.011	248 (11.6)	122 (11.8)	0.005
Liver disease	659 (31.0)	356 (34.3)	0.071	683 (32.1)	335 (32.3)	0.004
CHA_2_DS_2_-VASc score			0.137			0.005
Mean (SD)	3.32 ± 1.71	3.56 ± 1.79		3.4 ± 1.74	3.41 ± 1.73	
Median (IQR)	3 (2–4)	3 (2–5)		3 (2–5)	3 (2–5)	
Modified HAS-BLED			0.138			0.007
Mean (SD)	3.18 ± 0.94	3.31 ± 0.93		3.23 ± 0.94	3.24 ± 0.93	
Median (IQR)	3 (3–4)	3 (3–4)		3 (3–4)	3 (3–4)	
Concomitant medications						
RAAS inhibitors	1820 (85.5)	861 (83.0)	0.070	1822 (85.6)	860 (83.0)	0.071
Beta-blockers	1809 (85.0)	877 (84.5)	0.014	1811 (85.1)	877 (84.6)	0.011
Calcium channel blockers	1493 (70.2)	745 (71.8)	0.035	1506 (70.8)	737 (71.1)	0.007
Loop diuretics	1174 (55.2)	606 (58.4)	0.064	1188 (55.8)	591 (57.0)	0.023
Statins	1819 (85.5)	906 (87.3)	0.052	1826 (85.7)	905 (87.3)	0.046
NSAID	1390 (65.3)	696 (67.1)	0.036	1405 (66.0)	683 (65.9)	0.002
Proton pump inhibitors	782 (36.8)	405 (39.0)	0.046	800 (37.6)	387 (37.3)	0.005
Antithrombotic therapy						
OAC type			0.271			0.001
Warfarin	1320 (62.0)	505 (48.7)		1226 (57.6)	596 (57.5)	
DOAC	808 (38.0)	533 (51.4)		903 (42.4)	440 (42.5)	
DOAC dose			0.205			0.283
Standard dose DOAC	220 (27.2)	196 (36.8)		236 (26.2)	173 (39.4)	
Reduced dose DOAC	588 (72.78)	337 (63.2)		666 (73.8)	267 (60.6)	
DOAC type						
Rivaroxaban	327 (15.4)	209 (20.1)		364 (17.1)	173 (16.7)	
Dabigatran	179 (8.4)	114 (11.0)		198 (9.3)	97 (9.3)	
Apixaban	190 (8.9)	137 (13.2)		214 (10.1)	110 (10.6)	
Edoxaban	112 (5.3)	73 (7.0)		126 (5.9)	61 (5.9)	
Antiplatelet agent type						
Aspirin	1110 (52.2)	0(0)		1098 (51.6)	0(0)	
Clopidogrel	1015 (47.7)	0(0)		1028 (48.3)	0(0)	
Prasugrel or ticagrelor	3 (0.1)	0(0)		3 (0.1)	0(0)	

IQR, interquartile ranges; DOAC, direct oral anticoagulant; IPTW, inverse probability of treatment weighting; NSAID, non-steroidal anti-inflammatory drug; OAC, oral anticoagulant; RAAS, renin-angiotensin-aldosterone system; SD, standard deviation; SE, systemic embolism; TIA, transient ischemic attack.

### Effectiveness, safety, and net clinical benefit of oral anticoagulant plus single antiplatelet therapy vs. oral anticoagulant monotherapy in patients 1-year after percutaneous coronary intervention

**FIGURE 2:**
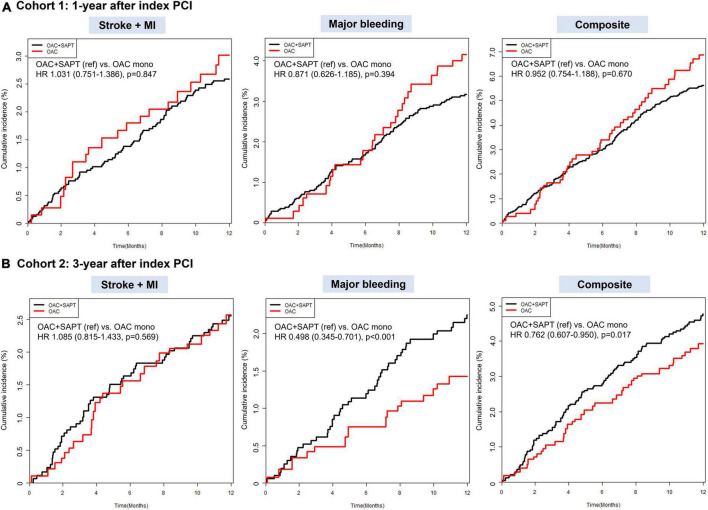
Weighted cumulative incidence curves for ischemic stroke/myocardial infarction, major bleeding, and composite clinical outcome: OAC plus SAPT vs. OAC monotherapy. **(A)** Cohort 1: patients with AF who underwent PCI, 1-year after index PCI. **(B)** Cohort 2: patients with AF who underwent PCI, 3-year after index PCI. HR, hazard ratio; OAC, oral anticoagulant; SAPT, single antiplatelet agent.

**FIGURE 3:**
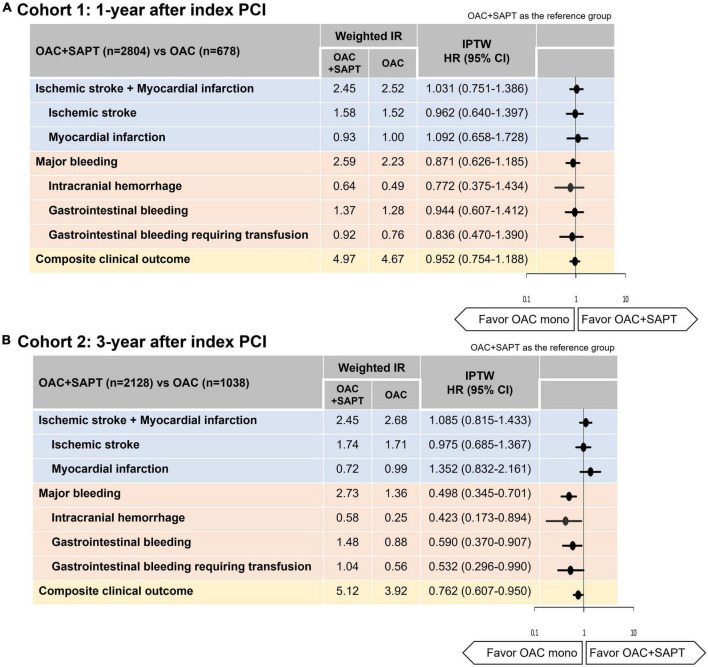
Hazard ratios of ischemic stroke, myocardial infarction, major bleeding, and composite clinical outcome: OAC plus SAPT vs. OAC monotherapy. **(A)** Cohort 1: patients with AF who underwent PCI, 1-year after index PCI. **(B)** Cohort 2: patients with AF who underwent PCI, 3-year after index PCI. IR, 100 person-years. CI, confidence interval; HR, hazard ratio; IR, incidence rate; IPTW, inverse probability of treatment weighting; OAC, oral anticoagulant; SAPT, single antiplatelet agent.

A median follow-up duration of cohort 1 was 2.3 (IQR, 1.2–4.2) years. Crude incidence rates of clinical outcomes and unadjusted HRs for clinical outcomes are presented in Supplementary Table 2. Figure 2A showed weighted cumulative incidence curves of effectiveness, safety, and composite clinical outcomes of cohort 1. Weighted incidence rates and weighted HRs are presented in Figure 3A. After IPTW, OAC monotherapy and OAC plus SAPT showed comparable risks for a composite of ischemic stroke and myocardial infarction, major bleeding, and composite clinical outcomes (Figures 2A, 3A). OAC monotherapy and OAC plus SAPT did not show any significant differences for the individual components of the effectiveness and safety outcomes.

### Effectiveness, safety, and net clinical benefit of oral anticoagulant plus single antiplatelet therapy vs. oral anticoagulant monotherapy in patients with 3-year after percutaneous coronary intervention

A median follow-up duration of cohort 2 was 2.5 (IQR, 1.3–4.2) years. Crude incidence rates of clinical outcomes and unadjusted HRs for clinical outcomes are presented in Supplementary Table 2. Figure 2B showed weighted cumulative incidence curves of effectiveness, safety, and composite clinical outcomes of cohort 2. Weighted incidence rates and weighted HRs are presented in Figure 3B. In cohort 2 with 3-year after PCI, OAC monotherapy and OAC plus SAPT showed a comparable risk for a composite of ischemic stroke and myocardial infarction, however, OAC monotherapy was associated with a lower risk of composite clinical outcomes (HR 0.762, 95% CI 0.607–0.950), mainly driven by a reduction of major bleeding risk (HR 0.498, 95% CI 0.345–0.701) compared to OAC plus SAPT (Figures 2B, 3B).

For each component of effectiveness and safety outcomes, OAC monotherapy and OAC plus SAPT group showed comparable risks for both ischemic stroke and myocardial infarction (Figure 3B). OAC monotherapy was associated with lower risks of intracranial hemorrhage, gastrointestinal bleeding, and gastrointestinal bleeding requiring transfusion than OAC plus SAPT (Figure 3B).

### Sensitivity analyses

Multivariable Cox analyses showed consistent results with the IPTW analyses in two cohorts (Supplementary Table 2).

## Discussion

**FIGURE 4:**
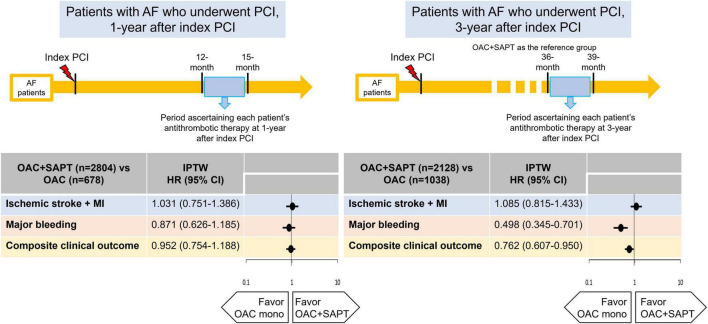
Hazard ratios of ischemic stroke, myocardial infarction, major bleeding, and composite clinical outcome: OAC plus SAPT vs. OAC monotherapy. AF, atrial fibrillation; CI, confidence interval; HR, hazard ratio; IPTW, inverse probability of treatment weighting; MI, myocardial infarction; OAC, oral anticoagulant; PCI, percutaneous coronary intervention; SAPT, single antiplatelet agent.

In this nationwide population-based observational study, our principal findings are as follows: (1) a substantial proportion of AF patients who had been receiving PCI for more than a year was prescribed OAC plus SAPT rather than OAC monotherapy; (2) among patients who had just passed 1 year after PCI, OAC monotherapy showed comparable risks for ischemic stroke, myocardial infarction, and major bleeding compared to OAC plus SAPT; (3) among patients 3 years after PCI, OAC monotherapy was associated with a lower risk of the composite clinical outcomes of ischemic stroke, myocardial infarction, and major bleeding than OAC plus SAPT, mainly driven by a lower risk of major bleeding. From these results, OAC monotherapy results in positive net clinical benefits by reducing bleeding risk in AF patients with sufficiently stable CAD after PCI (Figure 4). From the results of this study and previous clinical trials, OAC monotherapy would be the most reasonable option for patients with AF with stable CAD (1-year beyond PCI) as the current guidelines (1–4).

In a previous observational study based on the Danish nationwide cohort, warfarin-based OAC monotherapy was suggested as the most optimal antithrombotic therapy regimen in patients with stable CAD defined as 12 months from an acute coronary event (20). Compared to warfarin, single or dual antiplatelet therapy without anticoagulation was associated with increased risks of myocardial infarction, thromboembolism, death from the coronary event, and all-cause death. A combination of warfarin and single or dual antiplatelet therapy was related to the excessive bleeding risk compared to warfarin monotherapy.

Based on the consistent results of several observational studies (5), the guidelines have therefore advocated prescribing OAC monotherapy in AF patients 1 year following PCI as a Class IIa recommendation (21). However, the evidence generated through RCTs *per se* may be insufficient. The first RCT comparing OAC alone vs. OAC plus SAPT in patients with AF beyond 1 year after PCI, the OAC-ALONE trial, was reported (6). The median time from the last PCI was 4.4 (IQR 1.8–7.7) years in the OAC monotherapy group or 4.6 (IQR 2.4–7.4) years in OAC plus SAPT group, respectively. Among the total study population, only 25% were prescribed DOAC. Hence, the main results of the OAC-ALONE trial were inconclusive. More recently, the results of the AFIRE study, which included a large number of patients and used rivaroxaban as anticoagulation therapy, were published (7). This trial showed rivaroxaban monotherapy was significantly safer and more effective than rivaroxaban plus SAPT in patients with AF and stable CAD.

Despite the recommendations of the latest guidelines and updated evidence, a substantial proportion of patients with AF and stable CAD still do not receive guideline adherent antithrombotic therapy (22, 23). In contrast to the high rates of dual antiplatelet treatment, the overall rates of OAC were low after PCI in patients with AF. Since the emergence of DOACs, the usage of triple anti-thrombotic therapy in periprocedural antithrombotic regimens has shifted significantly, particularly in DOAC-based regimens. Regarding antithrombotic therapy 1 year after PCI, DAPT was more prevalent than OAC therapy. Also, OAC monotherapy 1 year after PCI was significantly lower than OAC plus SAPT therapy even in the DOAC era. In clinical practice, most patients with AF who underwent PCI continued to receive antiplatelet agents beyond 1-year post-PCI (23). This could be seen as a reflection of physicians’ preference for continuing to utilize the antiplatelet therapy in patients undergone PCI while omitting anticoagulation therapy because of the concern of excessive bleeding.

There have been two recent conflicting observational studies for patients with AF who underwent PCI beyond 1-year (8, 9). In a previous study including patients with AF who were at “early” stable period from PCI (immediate after 1-year), OAC plus SAPT seemed to be more effective than OAC monotherapy, without a difference in safety (8). In another previous study enrolled AF patients who were stable for more than 1-year after PCI, the mean time difference between the last PCI and the index date was 24 ± 18 months (9). OAC monotherapy showed similar efficacy to OAC plus SAPT and was associated with a lower risk of hospitalization due to bleeding compared to OAC plus SAPT. Neither net clinical benefit nor survival benefit of OAC monotherapy was documented.

Considering the results of previous studies and the trade-off of ischemic risk and bleeding risk after PCI (8–10, 24), the clinical benefits of OAC monotherapy over OAC plus SAPT may differ depending on how long it has elapsed since a year from PCI. However, there have been no studies attempting to analyze whether the benefit of treatment varies with the elapsed time after PCI in RCTs or observational studies. Recently, a *post-hoc* analysis of AFIRE study including patients who had undergone PCI has been reported which showed that in the PCI subgroup, the main results were consistently observed that rivaroxaban monotherapy was associated with lower risks of the primary efficacy and safety endpoints, compared to combination therapy (25). The median time from PCI to index date was 48 (IQR, 21–91) months, and most were more than 24 months after PCI. When analyzing the efficacy and safety endpoints over time after PCI, the differences in efficacy endpoints were not significant according to the time after PCI; however, in terms of safety endpoint, the longer the time elapsed after PCI, the more the OAC monotherapy benefits were accentuated compared to OAC plus SAPT. Overall, the net clinical benefit also became more evident with the longer time between PCI and enrollment. Our study showed consistent results through a large real-world observational cohort that the benefit of OAC monotherapy is more certain to reduce bleeding risk in patients with AF that are sufficiently stable after PCI.

While two RCTs have been reported (6, 7), more evidence is still needed for AF patients with stable CAD, and the results of the EPIC-CAD trial (NCT03718559), are awaited (26).

### Study limitations

First, there is a possibility of residual confounding, although we ascertained available variables and matched the balance between the two treatment groups. Among possible confounders, these data did not include information about the characteristics and numbers of coronary stents, the complexity of PCI procedure, and the presence of remaining significant coronary lesions. Second, this study is an observational study, which would include more comprehensive patients than RCTs in which patients are highly selected, but patients who died within 1 or 3 years or who received repeated PCI were excluded from the study design. However, if a physician considers prescribing patients without additional coronary events for several years after PCI, our data can be applied practically. Third, OAC monotherapy and OAC plus SAPT do not represent the majority of prescriptions in AF patients with stable CAD in Korea, who are often prescribed with antiplatelet agents only (22, 23). Therefore, the number of study subjects is limited, and it should be considered when interpreting the results that patients who received OAC prescriptions in real-world practice were selected by physicians. Fourth, the Korean HIRA database did not include laboratory findings such as serum creatinine. Therefore, to indirectly measure renal dysfunction, we included “renal diseases” as one of the baseline covariates defined using the operational definition adopted in previous observational studies based on the claims database (14, 22, 23). Fifth, among DOAC users in OAC plus SAPT group, a higher proportion of patients were prescribed reduced dose DOAC than those in the OAC monotherapy group. In previous observational studies and even in the RCT (6, 9), reduced dose DOAC was preferred in OAC plus SAPT group. In this dataset, patients’ body weight and creatinine clearance were not available, thus, DOAC dosing adherence could not be evaluated. Notwithstanding the higher proportion of reduced dose DOAC in the OAC plus SAPT group than in the OAC monotherapy group, a combination of OAC and SAPT still showed a higher risk of bleeding than OAC monotherapy. Sixth, two types of antiplatelet agents (aspirin and clopidogrel) were prescribed for the most of patients in the OAC plus SAPT group. Although which antiplatelet agents are better than others also can be an important question for clinical practice, the primary objective of this study was the comparison between OAC and OAC plus SAPT in patients with AF and stable CAD. The number of the study population was not sufficient to explore the better antiplatelet type or the better OAC type for these populations. Further clinical or observational studies are needed to answer this question.

## Conclusion

Oral anticoagulant monotherapy may be a comparable choice for patients with AF and stable CAD compared to OAC plus SAPT. In patients with stable CAD more than 3-year after index PCI, OAC monotherapy would be a better choice, being associated with less major bleeding and a positive net clinical benefit.

## Data availability statement

The raw data supporting the conclusions of this article will be made available by the authors, without undue reservation.

## Ethics statement

The studies involving human participants were reviewed and approved by the Institutional Review Board of the Seoul National University Hospital. Written informed consent for participation was not required for this study in accordance with the national legislation and the institutional requirements.

## Author contributions

S-RL and J-HJ: conceptualization, data curation, formal analysis, investigation, methodology, resources, software, validation, visualization, writing – original draft, figures and tables generation, and writing – review and editing. E-KC: conceptualization, formal analysis, investigation, methodology, resources, validation, funding acquisition, project administration, supervision, and writing – review and editing. S-WL, SK, and J-SP: conceptualization, data curation, formal analysis, investigation, methodology, resources, software, and validation. JK, K-DH, KP, SO, and GL: conceptualization, investigation, methodology, supervision, and writing – review and editing. All authors contributed to the article and approved the submitted version.
