# Supplementary material for: Net clinical benefit of antithrombotic therapy for atrial fibrillation patients with stable coronary artery disease
Source: Front Cardiovasc Med. 2022 Aug 22;9:991293. doi: 10.3389/fcvm.2022.991293 (PMC9441665; doi:10.3389/fcvm.2022.991293)

**Online supplementary Materials**

**Online Tables**

**Online Figures**

**Online Tables**

**Supplementary Table 1. Definitions of covariates and clinical outcomes**

| **Diagnosis** | **ICD-10-CM code and definition** | **Diagnostic definition** |
| --- | --- | --- |
| **Inclusion/exclusion criteria** |  |  |
| **Atrial fibrillation** | I48.0-48.4, I48.9 | Admission or outpatient department≥1 |
| **Valvular atrial fibrillation** | I05.0, I05.2, I05.9, Z95.2-Z95.4 |  |
| **Prior PCI** | Procedure codes M6551, M6552, M6561, M6562, M6563, M6564, M6571, M6572 | Admission and procedure codes |
| **Comorbidities** |  |  |
| **Hypertension** | I10-I13, I15; and minimum 1 prescription of anti-hypertensive drug (thiazide, loop diuretics, aldosterone antagonist, alpha-/beta-blocker, calcium-channel blocker, angiotensin-converting enzyme inhibitor, angiotensin II receptor blocker). | Admission≥1 or outpatient department≥2 |
| **Diabetes mellitus** | E11-E14; and minimum 1 prescription of anti-diabetic drugs (sulfonylureas, metformin, meglitinides, thiazolidinediones, dipeptidyl peptidase-4 inhibitors, α-glucosidase inhibitors and insulin). | Admission≥1 or outpatient department≥2 |
| **Dyslipidemia** | E78 | Admission or outpatient department≥1 |
| **Heart failure** | I50 | Admission or outpatient department≥1 |
| **Prior myocardial infarction** | I21, I22 | Admission or outpatient department≥1 |
| **Prior ischemic stroke/transient ischemic attack/systemic embolism** | I63, I64, G45, I74 | Admission or outpatient department≥1 |
| **Prior intracranial hemorrhage** | I60-62 | Admission or outpatient department≥1 |
| **Prior gastrointestinal bleeding** | I85, K22.1, I22.8, K25.0, K25.2, K25.4, K25.6, K26.0, K26.2, K26.4, K26.6, K27.0, K27.2, K27.4, K27.6, K28.0, K28.2, K28.4, K28.6, K29.0, K31.8, K92.0, K92.1, K92.2, K55.2, K57.0, K57.1, K57.2, K57.3, K57.4, K57.5, K57.8, K57.9, K62.5, K66.1v | Admission or outpatient department≥1 |
| **Peripheral artery disease** | I70, I73 | Admission or outpatient department≥2 |
| **Renal disease** | I13.1, N03, N05, N10-N19, Z49, Z94.0, Z99.2 | Admission or outpatient department≥1 |
| **Liver disease** | K70, K72-K76, K71.3-K71.7 | Admission or outpatient department≥1 |
| **Scores** |  |  |
| **CHA_2_DS_2_-VASc score** | Heart failure (1 point), hypertension (1 point), age ≥75 years (2 points), diabetes (1 point), previous stroke/systemic embolism/transient ischemic attack (2 points), vascular disease (prior MI or PAD, 1 point) and female sex (1 point) | |
|  |  |  |
| **Modified HAS-BLED score*** | Hypertension (1 point), liver disease (1 point), renal disease (1 point), stroke history (1 point), bleeding history (1 point), age>65 years (1 point) and drug (concomitant use of NSAID or antiplatelet agent, 1 point) | |
| **Clinical outcome** |  |  |
| **Ischemic stroke** | I63, I64 | Primary diagnosis, admission≥1 (≥3 days) and brain imaging (CT or MRI) ≥1 |
| **Myocardial infarction** | I21, I22 | Primary diagnosis, admission≥1 |
| **Major bleeding** | Intracranial hemorrhage or gastrointestinal bleeding or extracranial/unclassified major bleeding | Each definition was described as below. |
| **Intracranial hemorrhage** | I60-62 | Primary diagnosis, admission≥1 (≥3 days) and brain imaging (CT or MRI) ≥1 |
| **Gastrointestinal bleeding** | I85, K22.1, I22.8, K25.0, K25.2, K25.4, K25.6, K26.0, K26.2, K26.4, K26.6, K27.0, K27.2, K27.4, K27.6, K28.0, K28.2, K28.4, K28.6, K29.0, K31.8, K92.0, K92.1, K92.2, K55.2, K57.0, K57.1, K57.2, K57.3, K57.4, K57.5, K57.8, K57.9, K62.5, K66.1 | Primary diagnosis, admission≥1 |
| **Gastrointestinal bleeding requiring transfusion** | I85, K22.1, I22.8, K25.0, K25.2, K25.4, K25.6, K26.0, K26.2, K26.4, K26.6, K27.0, K27.2, K27.4, K27.6, K28.0, K28.2, K28.4, K28.6, K29.0, K31.8, K92.0, K92.1, K92.2, K55.2, K57.0, K57.1, K57.2, K57.3, K57.4, K57.5, K57.8, K57.9, K62.5, K66.1 | Primary diagnosis, admission≥1 and RBC transfusion≥1 |
| **Extracranial/unclassified major bleeding** | D62, H05.2, H35.6, H43.1, J94.2, M25.0, R04 | Primary diagnosis and [admission≥1 or RBC transfusion] |
| **Composite clinical outcome** | Ischemic stroke + myocardial infarction + major bleeding | Each definition was described as above. |

Abbreviation: CT, computed tomography; MRI, magnetic resonance image; PCI, percutaneous coronary intervention; RBC, red blood cell.

**Supplementary Table 2. Univariable and multivariable hazard ratios for clinical outcomes of OAC+SAPT versus OAC monotherapy at 1-year and 3-year**

|  | Event number (Crude IR) | | Unadjusted HR  (95% CI) | p-value | HR* (95% CI) | p-value |
| --- | --- | --- | --- | --- | --- | --- |
|  | OAC+SAPT  (reference) | OAC monotherapy |  |  |  |  |
| At 1-year |  |  |  |  |  |  |
| Ischemic stroke + myocardial infarction | 220 (2.46) | 48 (2.53) | 1.033 (0.756-1.412) | 0.838 | 1.050 (0.766-1.440) | 0.760 |
| Ischemic stroke | 144 (1.59) | 30 (1.58) | 0.987 (0.666-1.462) | 0.946 | 1.022 (0.687-1.521) | 0.913 |
| Myocardial infarction | 85 (0.92) | 19 (0.97) | 1.053 (0.640-1.732) | 0.839 | 1.044 (0.631-1.726) | 0.867 |
| Major bleeding | 231 (2.59) | 44 (2.28) | 0.881 (0.638-1.217) | 0.442 | 0.896 (0.648-1.241) | 0.509 |
| Intracranial hemorrhage | 59 (0.64) | 10 (0.50) | 0.790 (0.404-1.545) | 0.491 | 0.812 (0.413-1.596) | 0.545 |
| Gastrointestinal bleeding | 125 (1.37) | 25 (1.28) | 0.937 (0.610-1.440) | 0.766 | 0.954 (0.619-1.471) | 0.831 |
| Gastrointestinal bleeding requiring transfusion | 84 (0.81) | 15 (0.76) | 0.833 (0.481-1.443) | 0.514 | 0.837 (0.481-1.456) | 0.528 |
| Composite clinical outcome | 424 (4.96) | 87 (4.72) | 0.956 (0.759-1.204) | 0.704 | 0.974(0.772-1.23) | 0.826 |
| At 3-year |  |  |  |  |  |  |
| Ischemic stroke + myocardial infarction | 145 (2.40) | 67 (2.60) | 1.066 (0.797-1.424) | 0.667 | 1.043 (0.777-1.400) | 0.779 |
| Ischemic stroke | 103 (1.68) | 43 (1.64) | 0.962 (0.674-1.374) | 0.833 | 0.934 (0.650-1.342) | 0.712 |
| Myocardial infarction | 45 (0.72) | 26 (0.97) | 1.325 (0.817-2.149) | 0.253 | 1.328 (0.813-2.170) | 0.257 |
| Major bleeding | 161 (2.68) | 40 (1.51) | 0.563 (0.398-0.796) | 0.001 | 0.528 (0.372-0.750) | <0.001 |
| Intracranial hemorrhage | 36 (0.58) | 8 (0.30) | 0.510 (0.237-1.099) | 0.085 | 0.482 (0.222-1.049) | 0.065 |
| Gastrointestinal bleeding | 88 (1.44) | 26 (0.97) | 0.668 (0.431-1.035) | 0.071 | 0.623 (0.400-0.972) | 0.037 |
| Gastrointestinal bleeding requiring transfusion | 62 (1.00) | 16 (0.60) | 0.589 (0.340-1.021) | 0.059 | 0.541 (0.310-0.945) | 0.030 |
| Composite clinical outcome | 291 (5.02) | 100 (3.99) | 0.786 (0.626-0.987) | 0.038 | 0.750 (0.596-0.945) | 0.014 |

* Adjusted for age, sex, CHA_2_DS_2_-VASc score, HAS-BLED score, hypertension, diabetes mellitus, dyslipidemia, heart failure, myocardial infarction, peripheral artery disease, prior stroke/transient ischemic attack/systemic embolism, prior intracranial hemorrhage, prior gastrointestinal bleeding, renal disease, liver disease and OAC type (warfarin or DOACs).

IR, per 100 person-years

Abbreviation: CI, confidence interval; HR, hazard ratio; IR, incidence rate; DOAC, direct oral anticoagulant.

**Online Figure**

**Supplementary Figure 1. Distribution of propensity scores before and after IPTW**

Abbreviation: IPTW, inverse probability of treatment weighting; OAC, oral anticoagulant; PCI, percutaneous coronary intervention; SAPT, single antiplatelet agent.


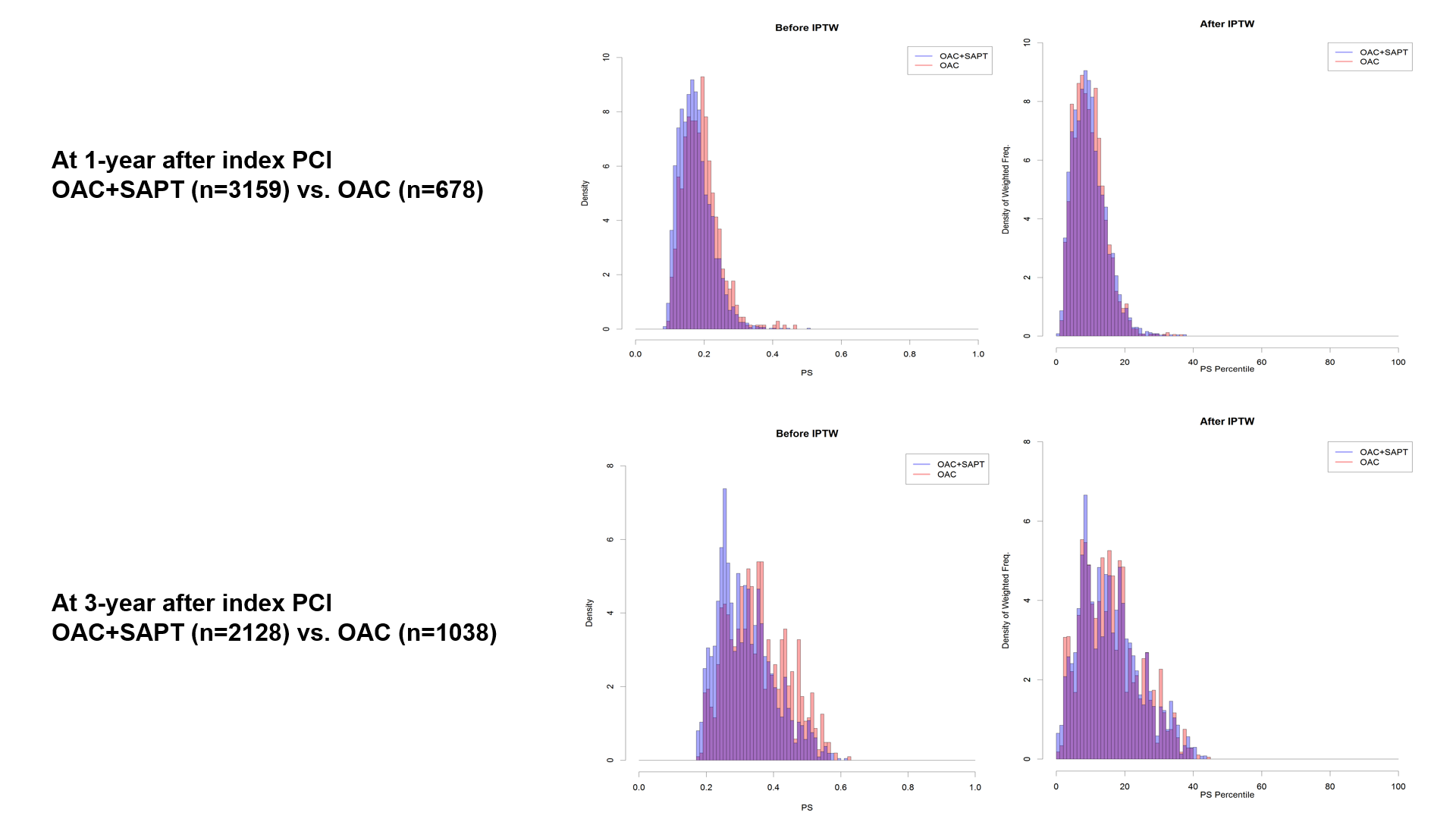

Supplement: Supplementary file 1 [file Data_Sheet_1.DOCX]
